# Enhancement of Cu-Cu Bonding Interfaces Through High Creep Rate in Nanocrystalline Cu

**DOI:** 10.3390/ma18163725

**Published:** 2025-08-08

**Authors:** Jian-Yuan Huang, Dinh-Phuc Tran, Kang-Ping Lee, Yi-Quan Lin, Emile Kuo, Tsung-Chuan Chen, Yao-Tsung Chen, Stream Chung, Chih Chen

**Affiliations:** 1 Institute of Pioneer Semiconductor Innovation, National Yang Ming Chiao Tung University, Hsinchu 300093, Taiwan; jyh.10@nycu.edu.tw; 2 Department of Materials Science and Engineering, National Yang Ming Chiao Tung University, Hsinchu 300093, Taiwan; trandinhphuc1508@gmail.com (D.-P.T.); michaelmichael1010@gmail.com (K.-P.L.); rex0199522@gmail.com (Y.-Q.L.); 3 Chemleaders Inc., Hsinchu 303036, Taiwan; emile_kuo@chemleader.com.tw (E.K.); tony@chemleader.com.tw (T.-C.C.); shima@chemleader.com.tw (Y.-T.C.); stream_chung@chemleader.com.tw (S.C.)

**Keywords:** 3D IC, Cu-Cu bonding, nanocrystalline Cu, interfacial voids, plan-view STEM

## Abstract

This study investigates the use of nanocrystalline Cu (NC-Cu) to suppress interfacial voids in low-temperature Cu-Cu bonding for 3D IC packaging. We quantitatively compared the void characteristics of electrodeposited NC-Cu (grain size ~89.3 nm) and (111)-oriented nanotwinned Cu (NT-Cu, ~621.8 nm) bonded at 200 °C. Plan-view STEM-HAADF analysis revealed that NC-Cu achieved a much lower void area ratio (1.8%) than NT-Cu (4.0%), attributed to its high grain boundary density, which enhances atomic diffusion and grain boundary migration. At 250 °C, typical Ostwald ripening was observed, with fewer but larger voids. However, a rise in total void area fraction suggests a competing mechanism—possibly new void nucleation at grain boundaries triggered by residual defects from the electroplating process. These results highlight the superior void-mitigating capability of NC-Cu under low thermal budgets.

## 1. Introduction

With the rapid advancement of cutting-edge technologies for artificial intelligence (AI) chips, the market demands on the performance, power consumption, and form factor of electronic components have become increasingly stringent [1,2,3]. Three-dimensional integrated circuit (3D IC) packaging technology has emerged as a key solution to continue Moore’s Law and achieve higher levels of integration [4,5]. Traditionally, vertical interconnects in 3D ICs rely on Sn-based solder. However, as the interconnect pitch continues to shrink, Sn-based solder faces severe reliability challenges, including intermetallic compound (IMC) formation, electromigration (EM), and Sn bridging, which limit its application in high-density packaging [6,7,8].

To address these bottlenecks, copper-to-copper (Cu-Cu) direct bonding is used. Compared to traditional solder, Cu-Cu bonding offers superior electrical and thermal conductivity, higher mechanical reliability, and enables the scaling of interconnect pitches to the sub-micron level [9]. Currently, this technology has been successfully implemented in commercial products. For instance, Sony Corp. has applied it to CMOS image sensors (CIS) to enhance pixel density and performance [10], while AMD has incorporated it into its 3D V-Cache technology via TSMC System on Integrated Chips (SoIC) platform, significantly boosting processor cache performance [11]. These successful commercialization cases fully demonstrate the immense potential and importance of this technology in the industry.

However, Cu-Cu direct bonding technology still faces a core challenge due to low self-diffusion rate, and therefore conventional processes typically require temperatures exceeding 300 °C to provide sufficient thermal energy to drive atomic diffusion [12]. Such a high thermal budget can cause damage to temperature-sensitive components or complex systems-on-chip (SoC) [13]. Therefore, achieving reliable bonding at low temperatures (<300 °C) is critical [14]. During low-temperature bonding, interfacial voids are commonly formed at the bonding interface. These voids significantly affect the bond quality [15], not only weakening the mechanical strength of the joint but also acting as initiation sites for EM failures, severely impacting the long-term reliability [16,17,18].

To achieve low-temperature bonding and suppress void formation, the academic community has primarily focused on two approaches: surface treatment and microstructure engineering. Surface treatment techniques, such as plasma activation [19], self-assembled monolayers (SAMs) passivation [20,21], and inert metal passivation layers [22], can effectively improve bonding quality but often increase process complexity and cost. In comparison, microstructure engineering of Cu itself is considered as a more fundamental and promising approach. Among various microstructures, nanotwinned Cu (NT-Cu) with high (111) surface ratio has been shown to effectively promote surface creep at low temperatures, owing to its excellent mechanical and electrical properties [23] and the high surface diffusivity of the (111) plane, thus facilitating bonding [24].

In recent years, nanocrystalline Cu (NC-Cu) has garnered widespread attention. Studies have indicated that the inherent thermal instability of NC-Cu is advantageous for atomic diffusion at low temperatures, showcasing its potential in low-temperature bonding [25,26]. Theoretically, NC-Cu contains a high density of random grain boundaries, which act as a network of highways for rapid atomic diffusion. Molecular dynamics (MD) simulations have pointed out that the high-energy, unstable grain boundaries in NC-Cu not only provide pathways for diffusion but also offer a strong thermodynamic driving force for atomic migration and void closure through the energy released during grain coarsening, making it theoretically superior to the relatively stable NT-Cu in void suppression [27]. In addition, recent experimental studies have confirmed that NC-Cu in confined SiO_2_ vias exhibits an enhanced thermal expansion that is two times larger than that of conventional coarse-grained Cu, an effect attributed to Coble creep, which is particularly beneficial for hybrid bonding processes relying on Cu expansion to achieve contact [28]. More recent studies have confirmed that the application of NC-Cu enables a more complete cross-interface bonding structure under a low thermal budget [29,30]. However, despite theoretical and circumstantial evidence pointing to the superiority of NC-Cu, there is still a lack of direct, quantitative comparative studies on the interfacial void morphology of NC-Cu under low-temperature bonding conditions to explicitly verify the practical effectiveness of NC-Cu in void mitigation.

Therefore, this study aims to fill this gap. We have successfully fabricated NC-Cu films with an average grain size of 89.3 nm and (111) NT-Cu films with an average grain size of 621.8 nm via electrodeposition. These films were then directly bonded at a low temperature of 200 °C and 250 °C. We employed plan-view transmission electron microscopy (TEM) to systematically quantify and analyze the size, number, and areal ratio of interfacial voids at the NC-Cu and NT-Cu joints. The results unequivocally demonstrate that the void density at the NC-Cu bonding interface was significantly lower than that at the NT-Cu, offering high-quality, high-reliability Cu-Cu interconnects.

## 2. Materials and Methods

In this study, Cu films were electroplated on Si wafers sputtered with a Ti/Cu seed layer in the electrolyte containing 0.8 M CuSO_4_, 1 M H_2_SO_4_, and 40 ppm Cl^−^, all purchased from Echo Chemical Co., Ltd. (Hsinchu, Taiwan). Two grain refiner additives, DP112 and DP115, supplied by Chemleader, Inc., Hsinchu, Taiwan, were added to the electrolyte to obtain nanoscale grains. For the control group of NT-Cu films, the electrolyte contained the 108C additive. The electrodeposition process was uniformly set at a current density of 12 ASD (A/dm^2^). The detailed equipment and parameters for the electrodeposition are described in Ref. [30]. Following Cu film deposition, all wafers were subjected to chemical mechanical planarization (CMP) to reduce the surface roughness *R*q. Cleaning was performed using standard organic solvents and citric acid. Direct Cu-Cu bonding was carried out in a vacuum environment using a WB-L3000 bonder (MATTECH, Hsinchu, Taiwan) under a downforce of 22 MPa for 1 h, at 200 °C or 250 °C. In addition, some NC-Cu bonded specimens were post-annealed at 250 °C for 1 h in a vacuum furnace to investigate the impact of thermal treatment on interfacial void formation. The selection of bonding temperatures in this study is based on both current industrial practices and future application scenarios. Specifically, 200 °C was chosen to reflect the temperature constraints of high bandwidth memory (HBM) technology, where low thermal budgets are critical to prevent damage to temperature-sensitive devices. In contrast, 250 °C was selected to represent higher-end bonding or post-bond annealing conditions that are sometimes adopted in advanced packaging processes. This temperature range enables a comparative investigation of how increased thermal budgets affect void evolution and grain boundary behavior at the Cu-Cu interfaces.

The surface roughness was measured using an atomic force microscope (AFM, D3100, VEECO, Plainview, NY, USA), with the scan rate and area set to 0.5 Hz and 5 × 5 µm^2^, respectively. To analyze the nanoscale microstructure, plan-view samples were prepared from the near-surface region of the NC-Cu specimens using a dual beam focused ion beam (DB-FIB, Helios 5 UX, Thermo Fisher Scientific Inc., Waltham, MA, USA). Subsequently, transmitted Kikuchi diffraction (TKD) scans were performed over an area of 3 µm × 3 µm using an electron backscattered diffraction (EBSD) detector (Oxford Instruments, Oxfordshire, UK). In contrast, the microstructure of the NT-Cu specimens was analyzed directly via EBSD in a Gemini 300 scanning electron microscope (SEM, Oberkochen, Germany). Both TKD and EBSD data were processed with orientation image mapping (OIM) software to quantify grain size and crystal orientation.

The morphology of the post-bonding cross-sectional interface was initially observed using FIB. For a more in-depth analysis of interfacial voids, plan-view transmission electron microscope (TEM) specimens containing the complete bonding interface were prepared using an in-situ lift-out technique with the FIB. The analysis of nanoscale voids was performed using a Talos F200X TEM (Thermo Fisher Scientific Inc., Waltham, MA, USA). Specifically, high-angle annular dark-field (HAADF) imaging in STEM mode was employed for void analysis. Leveraging the high atomic number (Z-contrast) sensitivity of this mode, voids can be precisely identified as black regions in the images, revealing their location, morphology, and size. For quantitative statistics on the voids, the HAADF images were first imported into ImageJ software (1.54g), where the void regions were marked as pure black to enhance contrast. The software was then used to calculate the area of each void. Subsequently, the equivalent diameter was derived from the area. EBSD is effective for crystallographic analysis, it is not suitable for detecting small voids. As voids are empty spaces, they produce no diffraction signal, and their small size often falls below the EBSD spatial resolution (~tens of nanometers). Additionally, sample surface preparation can obscure their boundaries. In contrast, plan-view STEM and HAADF imaging offer higher resolution and contrast, allowing direct and reliable visualization of nanoscale voids. Thus, TEM-based methods were employed in this study.

## 3. Results

**Figure 1:**
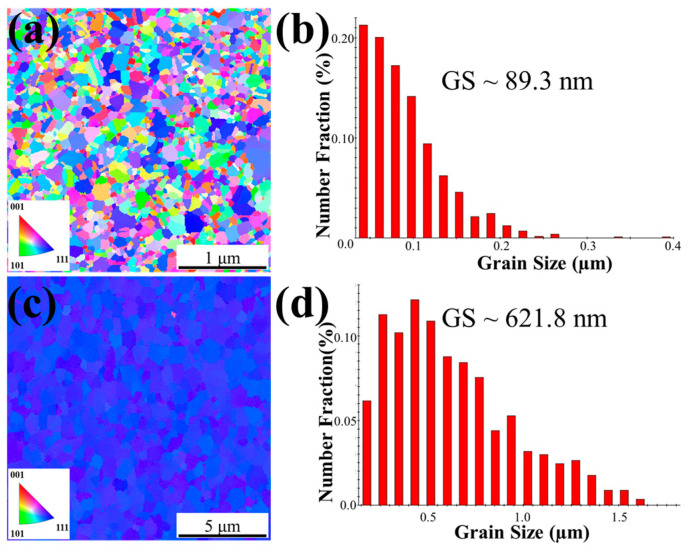
Plan-view TKD and EBSD images including OIM images, inverse pole figures and grain size distributions of the (**a**,**b**) NC-Cu and (**c**,**d**) NT-Cu films.

**Figure 2:**
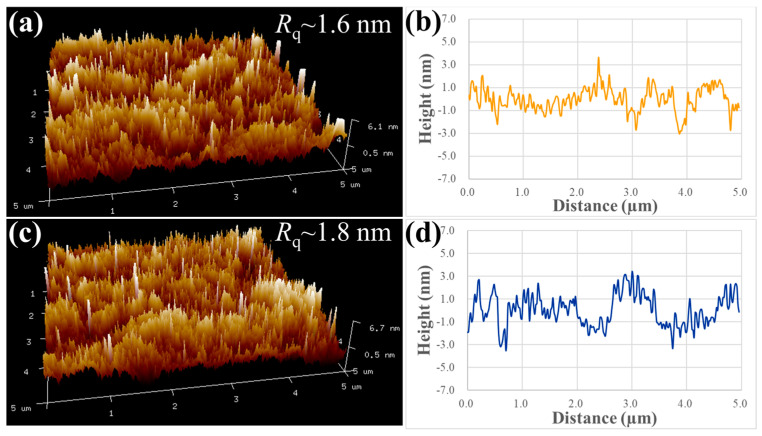
3D AFM images and corresponding surface profiles of Cu films after the CMP process: (**a**,**b**) NC-Cu; (**c**,**d**) NT-Cu. Surface roughness values (*R*q) are indicated in each image.

The microstructural analyses of the NC-Cu and NT-Cu surfaces prior to bonding are shown in Figure 1. The scanned EBSD area of the NC-Cu sample was 3 × 3 µm^2^. The TKD OIM image (Figure 1a) of the NC-Cu film exhibits a fine-grained structure with random crystallographic orientations. The corresponding grain size distribution in Figure 1b confirms its nanocrystalline nature, with an average grain size of approximately 89.3 nm and a grain boundary density of 40.5 µm^−1^. In contrast, the NT-Cu film (Figure 1c) shows significantly larger grains with a strong (111) texture, which was scanned in an area of 15 × 15 µm^2^. The grain size distribution in Figure 1d shows an average grain size of 621.8 nm and a grain boundary density of 4.8 µm^−1^. Generally, the NC-Cu film possesses a fine-grained, randomly oriented microstructure, whereas the NT-Cu film exhibits a coarse-grained structure with strong crystallographic texture. Notably, the grain boundary density of the NC-Cu was approximately 8 times larger than that of the NT-Cu. In addition to microstructural differences, the as-deposited NC-Cu exhibited a resistivity of 2.2 × 10^−6^ Ω·cm [30], slightly higher than that of the NT-Cu film (1.8 × 10^−6^ Ω·cm). This increase is attributed to the higher grain boundary density in NC-Cu, which enhances electron scattering. Figure 2 shows the 3D AFM images and surface profiles of two Cu films after CMP. The results indicate that both the NC-Cu film (Figure 2a,b) and the NT-Cu film (Figure 2c,d) exhibit highly smooth surfaces, with root-mean-square *R*q roughness values of approximately 1.6 nm and 1.8 nm, respectively. These low surface roughness values ensure that topography is not a dominant variable in the subsequent bonding experiments, allowing for a direct comparison of the influence of microstructural differences on bonding properties.

**Figure 3:**
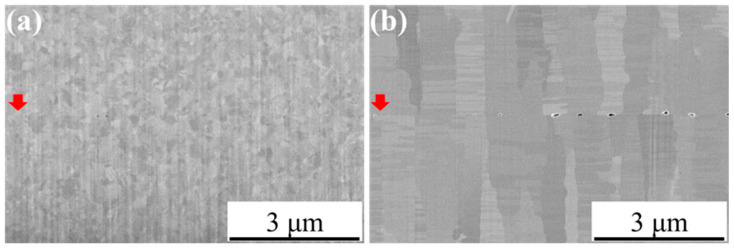
Cross-sectional images of the bonding interfaces acquired using the e-beam imaging function of the FIB system after milling. (**a**) NC-Cu bonded at 200 °C/22 MPa/1 h; (**b**) NT-Cu bonded under the same conditions. The bonding interfaces are marked with red arrows.

**Figure 4:**
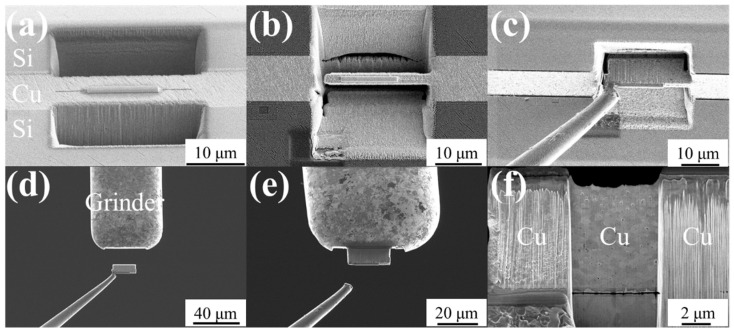
Plan-view TEM specimen preparation process for observing interfacial voids in the bonded Cu-Cu samples: (**a**) the top and bottom Si substrates were removed using FIB milling, (**b**) the Cu-Cu bonded region was thinned to an appropriate thickness, (**c**) the lamella was picked up using a micromanipulator probe, (**d**) the lamella was placed onto the grinder inside the FIB chamber, (**e**) the lamella was attached to the grinder surface by Pt deposition, (**f**) final thinning was performed on the central region using FIB until the thickness reaches approximately 80 nm for TEM observation.

The initial assessment of bonding quality was carried out using cross-sectional images of the Cu-Cu bonding interfaces obtained via the e-beam imaging function of the FIB system. As shown in Figure 3, both the NC-Cu sample in Figure 3a and the NT-Cu sample in Figure 3b were successfully bonded at 200 °C, with no apparent interfacial gaps. Some voids were observed along the bonding interfaces of both samples; however, the number of visible voids was relatively limited. Therefore, for a more comprehensive and quantitative evaluation of these interfacial defects, some plan-view TEM specimens were prepared using an in-situ pick-up method, as illustrated in Figure 4. The Si substrates above and below the bonding interface were first removed using Ga^+^ ion beam milling, followed by progressive thinning of the bonded Cu-Cu region. The lamella was then lifted out using a micromanipulator probe and attached to a grinder using Pt deposition. The region of interest was subsequently thinned further by FIB until the final thickness reached approximately 80 nm.

**Figure 5:**
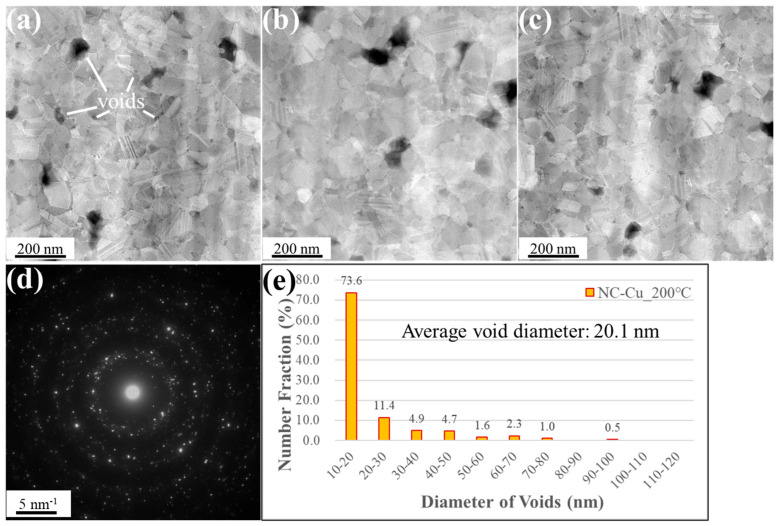
Plan-view STEM images of the NC-Cu specimen bonded under 200 °C/22 MPa/1 h. (**a**–**c**) High-magnification plan-view micrographs of the bonding interface, illustrating the interfacial voids (black areas). (**d**) Selected area diffraction pattern acquired from the interfacial region. (**e**) Statistical distribution of void sizes. Small voids are denoted by white lines.

**Figure 6:**
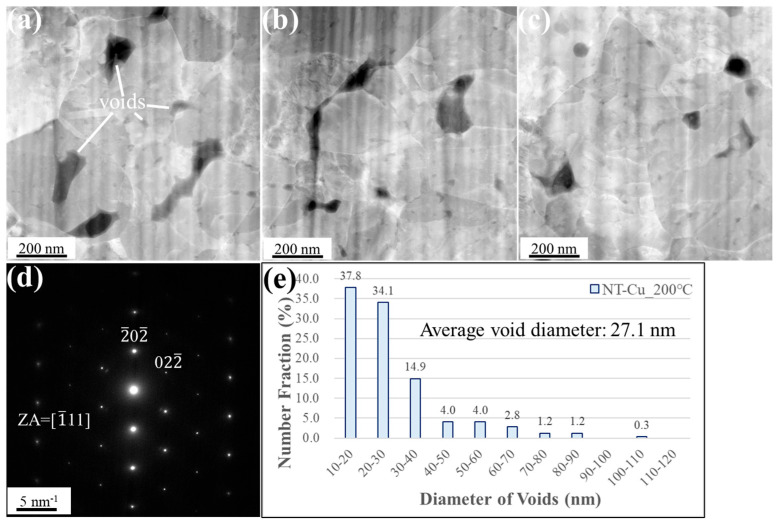
Plan-view STEM images of the NT-Cu bonded specimen under 200 °C/22 MPa/1 h. (**a**–**c**) High-magnification plan-view micrographs of the bonded interface, illustrating the presence of interfacial voids. (**d**) Selected area diffraction pattern acquired from the interfacial region. (**e**) Statistical distribution of void sizes. Small voids are denoted by white lines.

Figure 5 and Figure 6 show the plan-view STEM images of NC-Cu and NT-Cu samples bonded at 200 °C, respectively. The black/gray regions correspond to the interfacial voids. A distinct difference in void morphology was observed between the two samples. In Figure 5a–c, the NC-Cu interface features a large number of relatively small and circular voids, most of which were located along grain boundaries. The SAD pattern shown in Figure 5d displays concentric polycrystalline rings, indicating that the nanocrystalline structure was retained after bonding. The statistical distribution of void sizes, presented in Figure 5e, reveals that 73.6% of the voids fall within the 10–20 nm range, and 11.4% fall within 20–30 nm. In total, 85% of all voids had a diameter below 30 nm. Within the analyzed area of 10 µm^2^, the total void count was 386, with an average void diameter of 20.1 ± 14.2 nm, and a void area ratio of 1.8%. The detailed statistical results of each parameter are summarized in Table 1.

In contrast, Figure 6a–c show that the NT-Cu interface exhibited significantly larger and more irregularly shaped voids, mostly aligned along grain boundaries. The corresponding SAD pattern in Figure 6d shows sharp single-crystal diffraction spots, with a zone axis of [111], consistent with the initial high (111) ratio of the film. As shown in Figure 6e, 37.8% of the voids fall within the 10–20 nm range, while 34.1% fall within 20–30 nm. Overall, 71.9% of the voids had diameters below 30 nm, which was 13.1% lower than that of the NC-Cu sample. In the same 10 µm^2^ area, the NT-Cu sample contained 538 voids, with an average diameter of 27.1 ± 14.8 nm and a void area ratio of 4.0%. Although the NT-Cu sample contained more voids, their larger individual sizes resulted in a void area ratio that was more than twice that of NC-Cu. These results clearly demonstrate that under low-temperature bonding at 200 °C, the nanocrystalline structure of NC-Cu is significantly more effective at suppressing interfacial void formation compared to NT-Cu.

**Figure 7:**
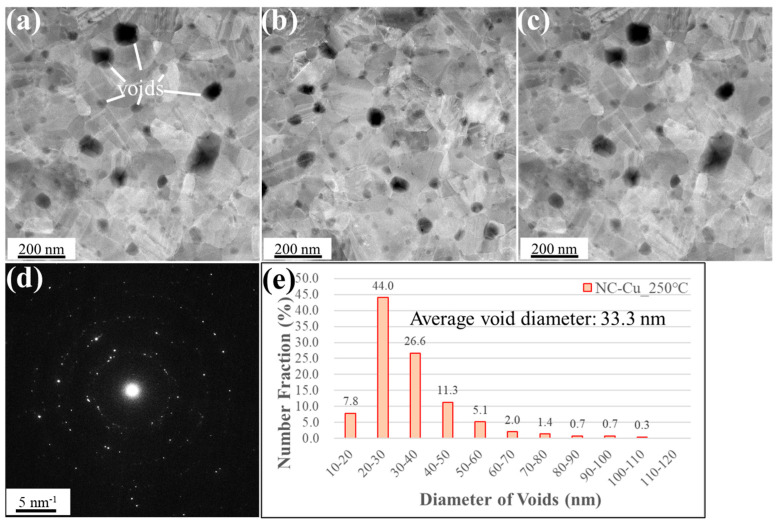
Plan-view STEM images of the NC-Cu bonded specimen under 250 °C/22 MPa/1 h. (**a**–**c**) High-magnification plan-view micrographs of the bonded interface, illustrating the presence of interfacial voids. (**d**) Selected area diffraction pattern acquired from the interfacial region. (**e**) Statistical distribution of void sizes. Small voids are denoted by white lines.

**Figure 8:**
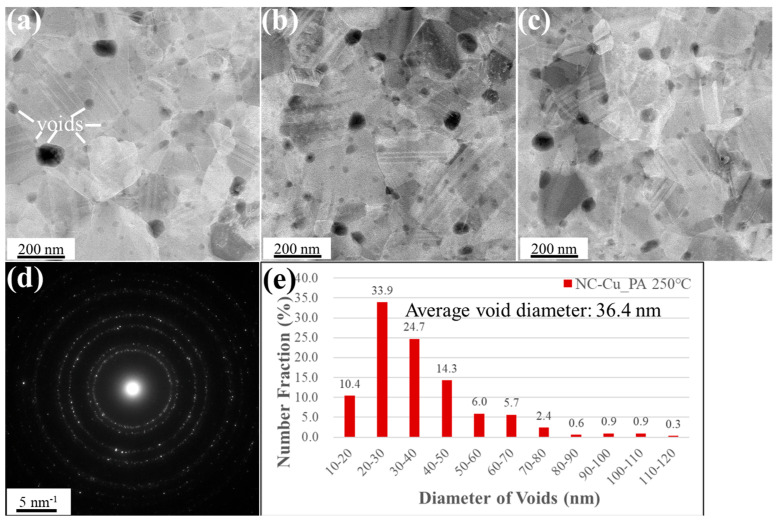
Plan-view STEM images of the NC-Cu bonded specimen under 200 °C/22 MPa/1 h and post-annealing at 250 °C for 1 h. (**a**–**c**) High-magnification plan-view micrographs of the bonded interface, illustrating the presence of interfacial voids. (**d**) Selected area diffraction pattern acquired from the interfacial region. (**e**) Statistical distribution of void sizes. Small voids are denoted by white lines.

To investigate the effect of thermal budget on void evolution in NC-Cu, two additional groups of samples were prepared. One group was bonded at 250 °C, while the other underwent post-annealing at 250 °C for 1 h after bonding at 200 °C. The corresponding plan-view STEM results are shown in Figure 7 and Figure 8. In Figure 7, when the bonding temperature was increased to 250 °C, a noticeable change in void morphology was observed at the NC-Cu bonding interface. The average void diameter increased to 33.3 ± 14.2 nm, while the total void count decreased to 366. The void area ratio increased to 3.8%, and the percentage of voids with diameters below 30 nm dropped significantly to 51.8%, compared to 85% under the 200 °C condition. In addition, the proportion of larger voids increased accordingly.

In the post-annealed sample shown in Figure 8, the average void diameter further increased to 36.4 ± 17.5 nm, while the total count dropped to 336. The void area ratio rose to 4.3%, and only 44.3% of voids had diameters below 30 nm. This trend is governed by the Ostwald ripening mechanism, in which smaller voids gradually dissolve and redeposit onto larger ones to minimize the overall interfacial energy. This result is aligned with the previous studies on void ripening kinetics in Cu-Cu bonding systems [17,18]. Figure 7d and Figure 8d show that the SAD patterns obtained under both conditions exhibit polycrystalline ring structures, indicating that the NC-Cu films retained their polycrystalline structure even after bonding or annealing at 250 °C for 1 h.

**Figure 9:**
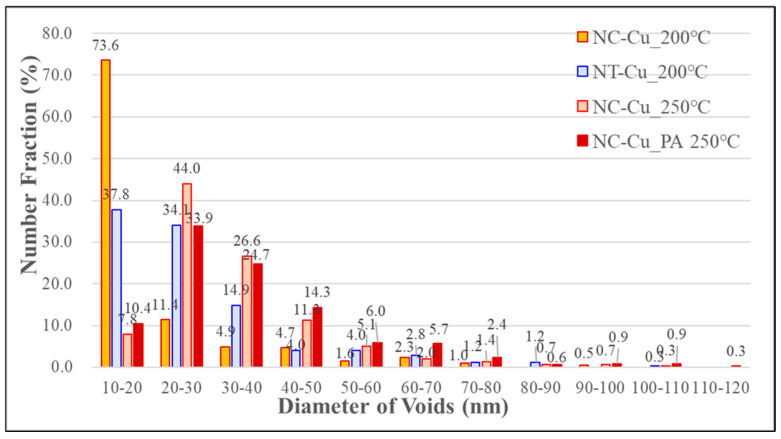
Comparative distribution of interfacial void diameters under different bonding conditions. NC-Cu bonded at 200 °C exhibits the highest proportion of small voids (10–20 nm), while the void size distribution broadens with increasing thermal budget, particularly after post-annealing at 250 °C.

To further illustrate the statistical differences among all bonding conditions studied, Figure 9 presents the number fraction distribution of void diameters. This comparative histogram highlights the shift in void population as a function of bonding temperature and post-annealing treatment, offering a clearer understanding of the thermal evolution of void characteristics. The NC-Cu bonded at 200 °C exhibits the highest fraction of small-sized voids in the 10–20 nm range. As the thermal budget increases, the void size distribution becomes broader, especially after post-annealing at 250 °C.

Notably, while the overall void count decreased as expected due to the Ostwald ripening mechanism, the total areal void ratio unexpectedly increased from 1.8% to 4.3% after post-annealing. This counterintuitive result suggests that other mechanisms may contribute to void formation. A plausible explanation is that thermal activation at 250 °C may cause voids to accumulate preferentially along regions with high grain boundary density, which serve as favorable sites for heterogeneous nucleation and thereby contribute to the increased void area fraction [31,32]. During high-temperature creep, voids are known to preferentially nucleate at triple junctions and high-angle grain boundaries due to their high interfacial energy and localized stress concentration [33]. In this study, the NC-Cu structure contains a dense network of randomly oriented grain boundaries, which may further amplify this effect and lead to the re-nucleation and growth of voids after thermal annealing. This phenomenon highlights the dual role of a high thermal budget. First, it promotes favorable microstructural evolution such as grain coarsening and interface reconstruction. On the other hand, if entrapped volatile impurities or defects are present in the as-deposited film, it may also trigger unintended defect formation mechanisms.

**Figure 10:**
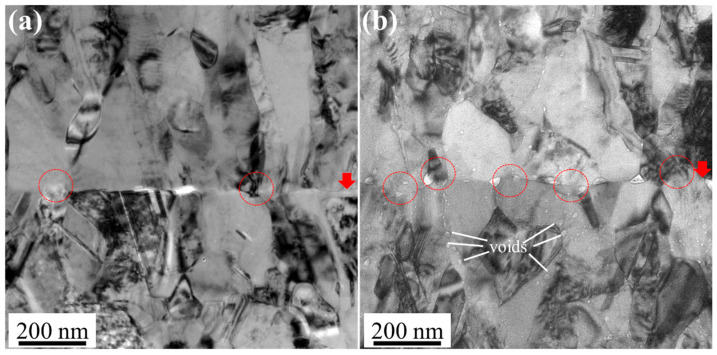
Cross-sectional bonding interface TEM images of the NC-Cu joints bonded at (**a**) 200 °C/22 MPa/1 h and (**b**) 250 °C/22 MPa/1 h. The red arrows indicate the bonding interfaces, while the red circles highlight the locations where the zig-zag microstructure was observed. Small voids located along grain boundaries are denoted by white lines.

Figure 10a,b show the cross-sectional TEM images of the bonding interfaces for NC-Cu and NT-Cu, respectively. The red arrows indicate the location of the bonding interface, while the red circles highlight the formation of zig-zag microstructures along the interface. As the bonding temperature was increased from 200 °C to 250 °C, the zig-zag microstructure became more pronounced. This feature provides direct evidence of grain boundary migration across the original bonding plane, which is critical for low temperature bonding. It is noted that the high grain boundary energy and the enhanced thermal expansion of NC-Cu offer a strong thermodynamic driving force for grain growth and interfacial reconstruction, even at relatively low temperatures [28]. However, as shown in Figure 10b, small voids were observed to nucleate along the grain boundaries at 250 °C, which aligns with the statistical trends in Figure 7. This suggests that elevated temperatures may promote the nucleation of new voids along the grain boundaries in NC-Cu, potentially driven by residual defect species originating from the electroplating process and facilitated by the high grain boundary density [32].

In addition, recent studies have emphasized that grain boundary mobility and void healing behavior in NC-Cu can be influenced by solute drag effects and boundary segregation, particularly under thermal exposure [29]. To address these challenges, strategies such as double-layer deposition or post-deposition treatments have been proposed to promote grain growth and enhance interfacial stability in NC-Cu systems [29,34,35]. Although such approaches are beyond the scope of this study, they are highly relevant for future process optimization and industrial integration.

Furthermore, previous shear testing on bonded specimens using the same NC-Cu [30] and NT-Cu [36] films demonstrated shear strengths exceeding 30 MPa and 20 MPa, respectively, with failures occurring within the Si dies rather than at the Cu–Cu interfaces. These results indicate that the interfaces are mechanically robust and further support the effectiveness of the proposed bonding process.

## 4. Discussion

**Figure 11:**
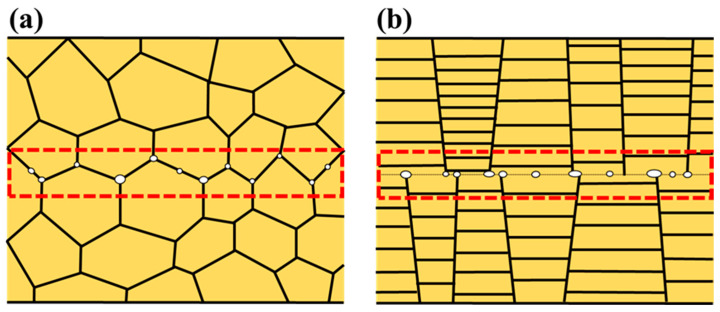
Schematic illustration of interfacial voids at the low-temperature bonded interface: (**a**) NC-Cu, (**b**) NT-Cu.

Under low-temperature bonding conditions, the void area ratio of the NC-Cu was significantly lower (1.8%) than that of the NT-Cu (4.0%). The mechanism behind this difference is illustrated in Figure 11. For NC-Cu, the dense and interconnected network of grain boundaries offers multiple diffusion pathways for atomic migration. Combined with the higher creep rate of NC-Cu, this facilitates Coble creep-driven mass transport, allowing atoms to migrate efficiently along the interface and fill voids. In contrast, for NT-Cu bonding, as shown in Figure 11b, the bonding interface remains relatively flat, and the lack of intersecting grain boundaries limits atomic diffusion. As a result, void removal is less effective, leading to a higher void density. In general, the enhanced atomic diffusion and creep rate in NC-Cu not only suppress void formation but also promote the formation of a robust and continuous zig-zag microstructure at the bonding interface, thereby contributing to a lower void area ratio.

To further validate the proposed creep mechanism, we quantitatively compared the grain size evolution at the bonding interface using plan-view STEM images. For the NC-Cu sample bonded at 200 °C for 1 h, the average grain size increased from 89.3 nm to 101.6 nm, indicating approximately 14% grain growth. In contrast, the NT-Cu sample exhibited negligible change, with the average grain size decreasing slightly from 621.8 nm to 618.5 nm. This selective grain growth in NC-Cu supports the occurrence of Coble creep-driven boundary migration, as finer-grained structures are known to exhibit enhanced boundary mobility and diffusion under thermal activation. These findings provide quantitative evidence for the higher creep rate of NC-Cu and reinforce the proposed diffusion-based mechanism for void reduction.

In addition to demonstrating a reduction in void area ratio and improved atomic diffusion behavior, the bonded NC-Cu samples have shown strong mechanical performance in prior shear testing. However, successful implementation of Cu-Cu bonding in advanced packaging also requires consideration of process scalability and integration compatibility. Recent studies [1,2,3,4] have highlighted issues such as wafer warpage, bonding interface coplanarity, and post-bond cleaning requirements, which pose significant challenges for scaling Cu-Cu bonding in high-volume manufacturing. While these aspects were beyond the scope of the current study, future work will focus on evaluating the process integration feasibility and addressing these engineering-level barriers.

## 5. Conclusions

In this study, we systematically investigated and compared the efficacy of NC-Cu and NT-Cu films in mitigating interfacial voids during low-temperature Cu-Cu direct bonding. Our key findings are summarized as follows:

Through the quantitative plan-view TEM analysis, we provided direct evidence that NC-Cu is significantly more effective at suppressing void formation than NT-Cu under low-temperature bonding. The void area ratio at the NC-Cu bonding interface (1.8%) was less than half of that observed at the NT-Cu counterpart (4.0%). The superior performance of NC-Cu is attributed to its high density of random grain boundaries and high creep rate. These boundaries act as high-diffusivity pathways, enhancing atomic transport and facilitating grain boundary migration. This leads to the efficient elimination of nascent voids and the formation of a robust zig-zag interfacial structure, even at low temperatures.

When the bonding thermal budget was increased to 250 °C, typical Ostwald ripening behavior was observed, characterized by a decrease in void count and an increase in average void size. However, this was accompanied by an increase in the total void area ratio, suggesting the presence of an additional void formation mechanism. It is speculated that residual defect species during the electroplating process, combined with the high grain boundary density, may promote the nucleation of new voids along the grain boundaries. This research demonstrates that employing a nanocrystalline microstructure is a highly effective strategy for creating reliable and low-defect Cu-Cu interconnects at low temperatures for the next-generation 3D IC packaging technology.

## Figures and Tables

**Table 1:** Average void diameter, total void count, and areal void ratio obtained from the plan-view STEM images of NC-Cu bonded specimens.

Condition	NC-Cu Bonded at 200 °C	NT-Cu Bonded at 200 °C	NC-Cu Bonded at 250 °C	NC-Cu Bonded at 200 °C with Post-Annealing at 250 °C/1 h
Average void diameter (nm)	20.1 ± 14.2	27.1 ± 14.8	33.3 ± 14.2	36.4 ± 17.5
Void counts under 10 μm^2^	386	538	366	336
Ratio of void area (%)	1.8	4.0	3.8	4.3

## Data Availability

The original contributions presented in this study are included in the article material. Further inquiries can be directed to the corresponding author.

## References

[1] Lau J.H. (2025). Current Advances and Outlooks in Hybrid Bonding. IEEE Trans. Compon. Packag. Manuf. Technol..

[2] Zhao Z.-H., Gao L.-Y., Liu Z.-Q. (2025). Review of Cu-Cu Direct Bonding Technology in Advanced Packaging. Nanotechnology.

[3] Lee Y.-G., McInerney M., Joo Y.-C., Choi I.-S., Kim S.E. (2024). Copper Bonding Technology in Heterogeneous Integration. Electron. Mater. Lett..

[4] Lau J.H. (2023). Recent Advances and Trends in Cu–Cu Hybrid Bonding. IEEE Trans. Compon. Packag. Manuf. Technol..

[5] Chen C., Yu D., Chen K.-N. (2015). Vertical Interconnects of Microbumps in 3D Integration. MRS Bull..

[6] Qiao Y., Ma H., Yu F., Zhao N. (2021). Quasi-in-Situ Observation on Diffusion Anisotropy Dominated Asymmetrical Growth of Cu-Sn IMCs under Temperature Gradient. Acta Mater..

[7] Ma T., Zhang S., Zhang Z., Zhao Y., Shao W., Huang J., Chen S., Ye Z., Wang W., Yang J. (2024). Investigation on Interfacial Compound Growth Kinetics in Sn-0.7Cu/Cu Solder Joint and Mechanism Analysis: Experiments and Molecular Dynamics Simulations. Mater. Charact..

[8] Zhang Z., Chen W., Hu X., Yi G., Chen B., Wang J., Jiang L., Jiang X., Li Q. (2024). Influence of Temperature Gradient Bonding on the Micromorphology and Shear Performance of Sn-Based Solder Joints: Experiments and First Principles Calculations. J. Manuf. Process..

[9] Lau J.H. (2024). State of the Art of Cu–Cu Hybrid Bonding. IEEE Trans. Compon. Packag. Manuf. Technol..

[10] Manda S., Matsumoto R., Saito S., Maruyama S., Minari H., Hirano T., Takachi T., Fujii N., Yamamoto Y., Zaizen Y. (2019). High-Definition Visible-SWIR InGaAs Image Sensor Using Cu-Cu Bonding of III-V to Silicon Wafer. Proceedings of the 2019 International Electron Devices Meeting (IEDM).

[11] Hu C.C., Chen M.F., Chiou W.C., Yu D.C.H. (2019). 3D Multi-Chip Integration with System on Integrated Chips (SoIC^TM^). Proceedings of the 2019 Symposium on VLSI Technology.

[12] Chen K.N., Fan A., Tan C.S., Reif R., Wen C.Y. (2002). Microstructure Evolution and Abnormal Grain Growth during Copper Wafer Bonding. Appl. Phys. Lett..

[13] Jang E.-J., Kim J.-W., Kim B., Matthias T., Park Y.-B. (2011). Annealing Temperature Effect on the Cu-Cu Bonding Energy for 3D-IC Integration. Met. Mater. Int..

[14] Rebhan B., Hingerl K. (2015). Physical Mechanisms of Copper-Copper Wafer Bonding. J. Appl. Phys..

[15] Tan C.S., Reif R., Theodore N.D., Pozder S. (2005). Observation of Interfacial Void Formation in Bonded Copper Layers. Appl. Phys. Lett..

[16] Shie K.-C., Hsu P.-N., Li Y.-J., Tran D.-P., Chen C. (2021). Failure Mechanisms of Cu–Cu Bumps under Thermal Cycling. Materials.

[17] Liu H.-C., Gusak A.M., Tu K.N., Chen C. (2021). Interfacial Void Ripening in Cu Cu Joints. Mater. Charact..

[18] Ong J.-J., Tran D.-P., Huang H.-J., Chiu W.-L., Yang S.-C., Wu W.-W., Chiang H.-H., Chen C. (2024). Evolutions of Interfacial Microstructures in Cu/SiO2 Hybrid Joints during Temperature Cycling Tests. J. Mater. Res. Technol..

[19] Kim T.H., Howlader M.M.R., Itoh T., Suga T. (2003). Room Temperature Cu–Cu Direct Bonding Using Surface Activated Bonding Method. J. Vac. Sci. Technol..

[20] Tan C.S., Lim D.F., Ang X.F., Wei J., Leong K.C. (2012). Low Temperature CuCu Thermo-Compression Bonding with Temporary Passivation of Self-Assembled Monolayer and Its Bond Strength Enhancement. Microelectron. Reliab..

[21] Maestre Caro A., Travaly Y., Beyer G., Tokei Z., Maes G., Borghs G., Armini S. (2013). Selective Self-Assembled Monolayer Coating to Enable Cu-to-Cu Connection in Dual Damascene Vias. Microelectron. Eng..

[22] Hong Z.-J., Liu D., Hu H.-W., Cho C.-I., Weng M.-W., Liu J.-H., Chen K.-N. (2022). Investigation of Bonding Mechanism for Low-Temperature Cu-Cu Bonding with Passivation Layer. Appl. Surf. Sci..

[23] Lu L., Shen Y., Chen X., Qian L., Lu K. (2004). Ultrahigh Strength and High Electrical Conductivity in Copper. Science.

[24] Liu C.-M., Lin H.-W., Huang Y.-S., Chu Y.-C., Chen C., Lyu D.-R., Chen K.-N., Tu K.-N. (2015). Low-Temperature Direct Copper-to-Copper Bonding Enabled by Creep on (111) Surfaces of Nanotwinned Cu. Sci. Rep..

[25] Wang Y., Huang Y.-T., Liu Y.X., Feng S.-P., Huang M.X. (2022). Thermal Instability of Nanocrystalline Cu Enables Cu-Cu Direct Bonding in Interconnects at Low Temperature. Scr. Mater..

[26] Jhan J.-J., Wataya K., Nishikawa H., Chen C.-M. (2022). Electrodeposition of Nanocrystalline Cu for Cu-Cu Direct Bonding. J. Taiwan Inst. Chem. Eng..

[27] Tatsumi H., Kao C.R., Nishikawa H. (2025). Atomistic Behavior of Cu-Cu Solid-State Bonding in Polycrystalline Cu with High-Density Boundaries. Mater. Des..

[28] Lin H.-E., Tran D.-P., Chiu W.-L., Chang H.-H., Chen C. (2024). Enhanced Thermal Expansion with Nanocrystalline Cu in SiO_2_ Vias for Hybrid Bonding. Appl. Surf. Sci..

[29] He C., Zhou J., Zhou R., Chen C., Jing S., Mu K., Huang Y.-T., Chung C.-C., Cherng S.-J., Lu Y. (2024). Nanocrystalline Copper for Direct Copper-to-Copper Bonding with Improved Cross-Interface Formation at Low Thermal Budget. Nat. Commun..

[30] Huang J.-Y., Tran D.-P., Lee K.-P., Lin Y.-Q., Kuo E., Chen T.-C., Chen Y.-T., Chung S., Chen C. (2025). Fabrication and Properties of High Thermal Stability Nanocrystalline Cu for Low Temperature Cu-Cu Bonding. J. Mater. Res. Technol..

[31] Tu K.N. (2003). Recent Advances on Electromigration in Very-Large-Scale-Integration of Interconnects. J. Appl. Phys..

[32] Yin L., Borgesen P. (2011). On the Root Cause of Kirkendall Voiding in Cu^3^Sn. J. Mater. Res..

[33] Noell P.J., Deka N., Sills R.B., Boyce B.L. (2022). Identifying the Microstructural Features Associated with Void Nucleation during Elevated-temperature Deformation of Copper. Fatigue Fract. Eng. Mater. Struct..

[34] Koju R.K., Mishin Y. (2021). The Role of Grain Boundary Diffusion in the Solute Drag Effect. Nanomaterials.

[35] Yamakov V., Moldovan D., Rastogi K., Wolf D. (2006). Relation between Grain Growth and Grain-Boundary Diffusion in a Pure Material by Molecular Dynamics Simulations. Acta Mater..

[36] Huang J.-Y., Chen C. (2024). Effect of (111) Surface Ratio on the Bonding Quality of Cu-Cu Joints. Proceedings of the 2024 74th Electronic Components and Technology Conference (ECTC).

